# Fractional Form of a Chaotic Map without Fixed Points: Chaos, Entropy and Control

**DOI:** 10.3390/e20100720

**Published:** 2018-09-20

**Authors:** Adel Ouannas, Xiong Wang, Amina-Aicha Khennaoui, Samir Bendoukha, Viet-Thanh Pham, Fawaz E. Alsaadi

**Affiliations:** 1 Department of Mathematics and Computer Science, University of Larbi Tebessi, Tebessa 12002, Algeria; 2 Institute for Advanced Study, Shenzhen University, Shenzhen 518060, Guangdong, China; 3 Department of Mathematics and Computer Sciences, University of Larbi Ben M’hidi, Oum El Bouaghi 04000, Algeria; 4 Electrical Engineering Department, College of Engineering at Yanbu, Taibah University, Medina 42353, Saudi Arabia; 5 Modeling Evolutionary Algorithms Simulation and Artificial Intelligence, Faculty of Electrical & Electronics Engineering, Ton Duc Thang University, Ho Chi Minh City, Vietnam; 6 Department of Information Technology, Faculty of Computing and IT, King Abdulaziz University, Jeddah 21589, Saudi Arabia

**Keywords:** discrete chaos, discrete fractional calculus, hidden attractors, approximate entropy, stabilization

## Abstract

In this paper, we investigate the dynamics of a fractional order chaotic map corresponding to a recently developed standard map that exhibits a chaotic behavior with no fixed point. This is the first study to explore a fractional chaotic map without a fixed point. In our investigation, we use phase plots and bifurcation diagrams to examine the dynamics of the fractional map and assess the effect of varying the fractional order. We also use the approximate entropy measure to quantify the level of chaos in the fractional map. In addition, we propose a one-dimensional stabilization controller and establish its asymptotic convergence by means of the linearization method.

## 1. Introduction

Over the last few decades and since the Hénon map was first proposed [1], discrete-time chaotic dynamical systems have received a great deal of attention from numerous disciplines due to their ability to model various natural phenomena [2]. Throughout the years, a variety of chaotic maps has been proposed and their dynamics investigated [3,4,5,6,7]. The dynamics and control of such systems have been widely investigated [8,9,10,11,12,13]. Recently, an interesting investigation was carried out in [14] on a rather general 2D map that can, under certain parameters, have no fixed points and possess hidden dynamics. The authors examined the stability of the fixed points and showed that the map exhibits rich dynamics and may in some instances have a very tiny basin of attraction. Note that the term hidden attractors first came about in the investigation of continuous chaotic systems [15,16,17]. They refer to attractors that do not contain the neighborhoods of the equilibria. The hidden attractor property plays important roles in science and engineering [18,19,20,21]. The existence of such attractors in many engineering applications is considered problematic and requires stabilization [22,23,24]. The authors considered a new system inspired by the logistic map and examined its bifurcation and hidden dynamics. The schematic approach proposed in [14] for studying such hidden dynamics has been picked up by researchers such as [25].

In recent years, with the growing advancement in the field of discrete fractional calculus, a few studies have emerged considering the dynamics, control and applications of fractional chaotic maps [26,27,28,29,30,31,32]. In addition, there are few works related to chaotic maps with hidden attractors [14,25]. Especially, research to date has not yet studied fractional chaotic maps without a fixed point. It should be noted that although the inception of fractional continuous calculus took place centuries ago, its discrete counterpart was not properly explored until recently [33]. The first definition of a fractional difference operator was made by Diaz and Olser in 1974 [34]. In fact, the vast majority of available literature on the subject was published in the last decade, including [35,36,37,38,39,40].

In this paper, we examine the dynamics the fractional version of the general map proposed in [14] by means of phase plots and bifurcation diagrams. It is noted that there is no fixed point in such a chaotic fractional map. There is no fractional-order chaotic map without fixed points reported in the literature. As far as we aware, this is the first time that a fractional-order chaotic map without fixed points has been investigated. We show that the fractional order has a major impact on the chaotic range and the shape of hidden attractors. We also use the approximate entropy measure to quantify the level of chaotic behavior present in the fractional map. By varying the fractional order, we show that it has an impact on the entropy. We also propose a one-dimensional stabilization controller that forces the system states to zero asymptotically. Throughout our analysis, we make use of numerical methods to confirm the findings.

## 2. The Fractional Map without Fixed Points

**Figure 1:**
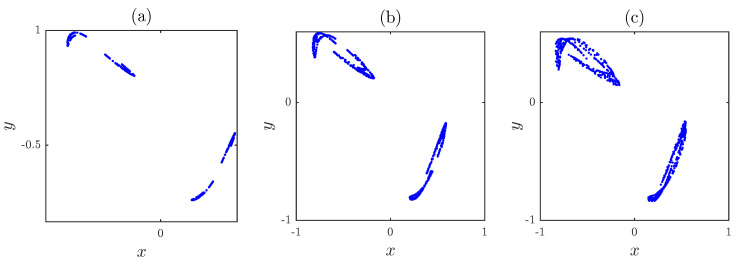
Phase plots of the map without a fixed point for: (**a**) $\left(a_{1},a_{2},a_{3},a_{4}\right)=\left(0.2,0.71,0.91,1.14\right)$ and $\left(x(0),y(0)\right)=\left(0.93,-0.44\right)$; (**b**) $\left(a_{1},a_{2},a_{3},a_{4}\right)=\left(0.51,1,1.51,0.74\right)$ and $\left(x(0),y(0)\right)=\left(-0.81,0.51\right)$; (**c**) $\left(a_{1},a_{2},a_{3},a_{4}\right)=\left(0.6,1,1.6,0.72\right)$ and $\left(x(0),y(0)\right)=\left(-0.26,0.18\right)$.

In this paper, we are interested in the dynamics, entropy and control of the fractional map based on the standard iterated map of the form:

(1) $$\left\{\begin{array}{c} x\left(n+1\right)=y\left(n\right),\\ y\left(n+1\right)=x\left(n\right)+a_{1}x^{2}\left(n\right)+a_{2}y^{2}\left(n\right)-a_{3}x\left(n\right)y\left(n\right)-a_{4}, \end{array}\right.$$

where $a_{1}$, $a_{2}$, $a_{3}$ and $a_{4}$ are some real-value parameters. This map was developed by Jiang et al. [14] as a variation of the original Hénon map [1]. They showed by means of analytical and numerical methods that hidden chaotic attractors exist in the map for certain values of the parameters $a_{1}$, $a_{2}$, $a_{3}$ and $a_{4}$. The phase-space portraits of the map with no fixed point are depicted in Figure 1 for three typical examples. This map has some interesting dynamics with hidden strange attractors. The authors showed that chaos exists in the map with different scenarios: no fixed point, a single fixed point and two fixed points. In the following, we develop a fractional chaotic map based on (1) to examine its dynamics and control.

Before we can state the fractional map we are concerned with, let us recall some important aspects of discrete fractional calculus. First of all, consider a generic function $X\left(t\right):N_{a}\to R$ where $N_{a}$ denotes the set of all discrete numbers starting from *a*, i.e., $N_{a}=\left\{a,a+1,a+2,\ldots \right\}$. Given a fractional difference number υ>0 and the function σ(s)=s+1, we define the υ-th fractional sum of $X\left(t\right)$ similar to [35] as:

(2) $$\Delta_{a}^{-\upsilon }X\left(t\right)=\frac{1}{\Gamma \left(\upsilon \right)}\sum_{s=a}^{t-\upsilon }{\left(t-\sigma \left(s\right)\right)}^{\left(\upsilon -1\right)}X\left(s\right),$$

for all $t\in N_{a+n-\upsilon }$ and with $t^{\upsilon }$ being the falling function defined in terms of the Gamma function Γ as:

(3) $$t^{\upsilon }=\frac{\Gamma \left(t+1\right)}{\Gamma \left(t+1-\upsilon \right)}.$$

With this in mind, we may define the υ-th Caputo type delta difference of $X\left(t\right)$ similar to [36] by:

(4) $${}^{C}\Delta_{a}^{\upsilon }X\left(t\right)=\Delta_{a}^{-(n-\upsilon )}\Delta^{n}X\left(t\right)=\frac{1}{\Gamma \left(n-\upsilon \right)}\sum_{s=a}^{t-\left(n-\upsilon \right)}{\left(t-\sigma \left(s\right)\right)}^{\left(n-\upsilon -1\right)}\Delta_{s}^{n}X\left(s\right),$$

where υ∉N is the fractional order, $t\in N_{a+n-\upsilon }$ and $n=\left[\upsilon \right]+1$.

Now, that we have stated the basics of discrete fractional calculus, we may start our analysis. System (1) can be rewritten in difference form as:

(5) $$\left\{\begin{array}{c} \Delta x\left(n\right)=y\left(n\right)-x\left(n\right),\\ \Delta y\left(n\right)=x\left(n\right)+a_{1}x^{2}\left(n\right)+a_{2}y^{2}\left(n\right)-a_{3}x\left(n\right)y\left(n\right)-a_{4}-y\left(n\right). \end{array}\right.$$

Then, using the Caputo difference operator ${}^{C}\Delta_{a}^{\upsilon }$ as defined in (4), we obtain the fractional version of the map for ${t\in N}_{a+1-\upsilon }$ and 0<υ≤1 as:

(6) $$\left\{\begin{array}{cc} {}^{C}\Delta_{a}^{\upsilon }x\left(t\right) & =y\left(t-1+\upsilon \right)-x\left(t-1+\upsilon \right),\\ {}^{C}\Delta_{a}^{\upsilon }y\left(t\right) & =x\left(t-1+\upsilon \right)+a_{1}x^{2}\left(t-1+\upsilon \right)+a_{2}y^{2}\left(t-1+\upsilon \right)\\ & \;-a_{3}x\left(t-1+\upsilon \right)y\left(t-1+\upsilon \right)-a_{4}-y\left(t-1+\upsilon \right). \end{array}\right.$$

From here on, we will refer to (6) as the fractional map. Our new fractional-order map belongs to a special class of dynamical systems with “hidden attractors”, which have received significant attention recently [19,24]. We believe that our work will assist researchers in further understanding systems with hidden attractors.

In order to examine the dynamics of the fractional map (6), we must develop a numerical formula for it. Let us recall an important theorem that defines the equivalent discrete integral equation corresponding to a generic fractional difference, which will enable us to obtain our numerical formula.

**Theorem** **1**  ([41])**.** *For the delta fractional difference equation:*

(7) $$\left\{\begin{array}{c} {}^{C}\Delta_{a}^{\upsilon }u\left(t\right)=f\left(t+\upsilon -1,u\left(t+\upsilon -1\right)\right),\\ \Delta^{k}=u_{k},\;n=\left[\upsilon \right]+1,\;k=0,1,\ldots ,n-1, \end{array}\right.$$

*the equivalent discrete integral equation can be obtained as:*

(8) $$u\left(t\right)=u_{0}\left(t\right)+\frac{1}{\Gamma \left(\upsilon \right)}\sum_{s=a+n-\upsilon }^{t-\upsilon }{\left(t-\sigma \left(s\right)\right)}^{\left(\upsilon -1\right)}f\left(s+\upsilon -1,u\left(s+\upsilon -1\right)\right),\;t\in N_{\alpha +n},$$

*where:*

(9) $$u_{0}\left(t\right)=\sum_{k=0}^{m-1}\frac{{\left(t-a\right)}^{k}}{k}\Delta^{k}u\left(a\right).$$

By applying Theorem 1, we can state the equivalent discrete integral form of (6) for $t\in N_{a+1}$ as:

(10) $$\left\{\begin{array}{cc} x\left(t\right)= & x\left(a\right)+\frac{1}{\Gamma \left(\upsilon \right)}\sum_{s=a+1-\upsilon }^{t-\upsilon }{\left(t-\sigma \left(s\right)\right)}^{\left(\upsilon -1\right)}\left(y\left(s+\upsilon -1\right)-x\left(s+\upsilon -1\right)\right),\\ y\left(t\right)= & y\left(a\right)+\frac{1}{\Gamma \left(\upsilon \right)}\sum_{s=a+1-\upsilon }^{t-\upsilon }{\left(t-\sigma \left(s\right)\right)}^{\left(\upsilon -1\right)}\left(x\left(s+\upsilon -1\right)+a_{1}x^{2}\left(s+\upsilon -1\right)\right.\\ & \;\left.+a_{2}y^{2}\left(s+\upsilon -1\right)-a_{3}x\left(s+\upsilon -1\right)y\left(s+\upsilon -1\right)-a_{4}-y\left(s+\upsilon -1\right)\right). \end{array}\right.$$

The reciprocal $\frac{{\left(t-\sigma \left(s\right)\right)}^{\left(\upsilon -1\right)}}{\Gamma \left(\upsilon \right)}$ is known as a discrete kernel function. For simplicity, we may choose:

(11) $$\frac{{\left(t-\sigma \left(s\right)\right)}^{\left(\upsilon -1\right)}}{\Gamma \left(\upsilon \right)}=\frac{\Gamma \left(t-s\right)}{\Gamma \left(\upsilon \right)\Gamma \left(t-s-\upsilon +1\right)}.$$

This leads to the following numerical formulas for a=0:

(12) $$\;\left\{\begin{array}{cc} x\left(n\right)=x\left(0\right) & +\frac{1}{\Gamma \left(\upsilon \right)}\sum_{j=1}^{n}\frac{\Gamma \left(n-j+\upsilon \right)}{\Gamma \left(n-j+1\right)}\left(y\left(j-1\right)-x\left(j-1\right)\right),\\ y\left(n\right)=y\left(0\right) & +\frac{1}{\Gamma \left(\upsilon \right)}\sum_{j=1}^{n}\frac{\Gamma \left(n-j+\upsilon \right)}{\Gamma \left(n-j+1\right)}\left(x\left(j-1\right)+a_{1}x^{2}\left(j-1\right)\right.\\ & \;\left.+a_{2}y^{2}\left(j-1\right)-a_{3}x\left(j-1\right)y\left(j-1\right)-a_{4}-y\left(j-1\right)\right), \end{array}\right.$$

These numerical formulas will allow us to plot phase-space portraits, bifurcation diagrams and error convergence plots throughout the remainder of this paper.

## 3. Chaotic Dynamics and Entropy Analysis

### 3.1. Chaotic Dynamics

**Figure 2:**
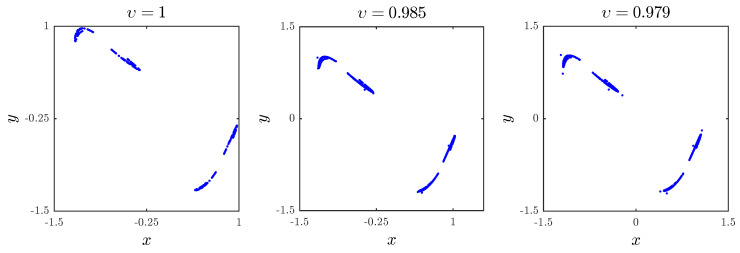
The chaotic attractor obtained with $\left(a_{1},a_{2},a_{3},a_{4}\right)=\left(0.2,0.71,0.91,1.14\right)$ and $\left(x(0),y(0)\right)=\left(0.93,-0.44\right)$ for different fractional orders υ.

Now that we have our fractional map (6) and the corresponding numerical formulas (12), let us study the map’s dynamics and chaotic behavior. First, we study the effect of the fractional order υ on the dynamics of the map for parameter values $\left(a_{1},a_{2},a_{3},a_{4}\right)=\left(0.2,0.71,0.91,1.14\right)$. Evaluating (12) for υ=1 and with some direct calculations, we can see that the resulting dynamics of the fractional map are identical to those of the classical one even though it has a discrete memory effect, i.e., the solution $x\left(n\right)$ depends on all previous values $x\left(0\right),x\left(1\right),\ldots ,x\left(n-1\right)$. With initial values $\left(x\left(0\right),y\left(0\right)\right)=\left(0.93,-0.44\right)$, Figure 2 shows the phase portrait of the fractional map for various values of the fractional order υ. We notice that as υ decreases, the trajectory $\left(x\left(t\right),y\left(t\right)\right)$ remains bounded, whereas when υ≤0.976, the chaotic behavior is delayed and the states of the fractional map diverge to infinity.

**Figure 3:**
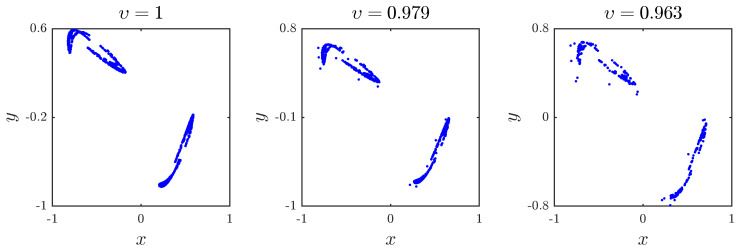
The chaotic attractor obtained with $\left(a_{1},a_{2},a_{3},a_{4}\right)=\left(0.51,1,1.51,0.74\right)$ and $\left(x(0),y(0)\right)=\left(-0.81,0.51\right)$ for different fractional orders υ.

Next, we set the parameters $\left(a_{1},a_{2},a_{3},a_{4}\right)$–$\left(0.51,1,1.51,0.74\right)$ and choose the initial values $\left(x\left(0\right),y\left(0\right)\right)=\left(-0.81,0.51\right)$. Figure 3 depicts the phase portraits of the fractional map for the three different fractional orders υ=1, υ=0.979 and υ=0.963. Similarly, when υ=1, the fractional map refers to the classical system. While 0.963≤υ≤1, the fractional map (6) exhibits a chaotic behavior, and when υ=0.962, we fall into an unbounded attractor.

**Figure 4:**
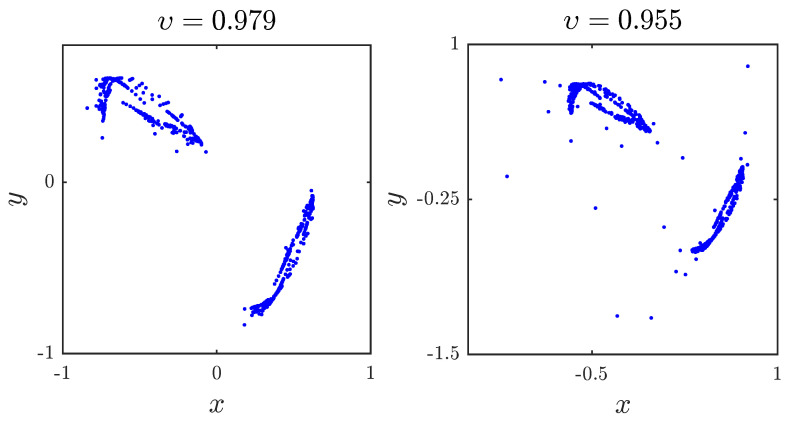
The chaotic attractor obtained with $\left(a_{1},a_{2},a_{3},a_{4}\right)=\left(0.6,1,1.6,0.72\right)$ and $\left(x(0),y(0)\right)=\left(-0.26,0.18\right)$ for different fractional orders υ.

Let us, now, consider the third set of parameters $\left(a_{1},a_{2},a_{3},a_{4}\right)=\left(0.6,1,1.6,0.72\right)$ with initial states $\left(x\left(0\right),y\left(0\right)\right)=\left(-0.26,0.18\right)$. As can be seen in Figure 4, the resulting trajectories of the fractional map vary with υ. When υ<0.95, chaos disappears completely.

**Figure 5:**
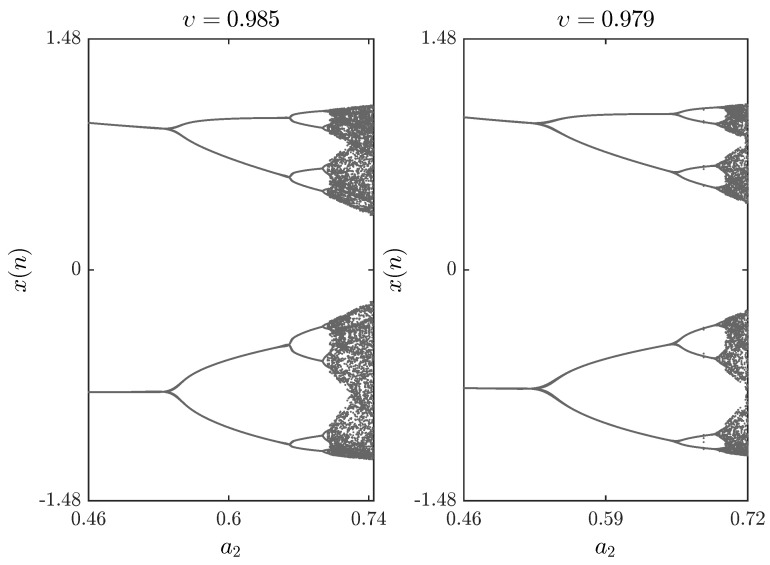
Bifurcation diagrams with $a_{2}$ as the critical parameter and $\left(a_{1},a_{3},a_{4}\right)=\left(0.2,0.91,1.14\right)$ and $\left(x(0),y(0)\right)=\left(0.93,-0.44\right)$ for different fractional orders υ.

**Figure 6:**
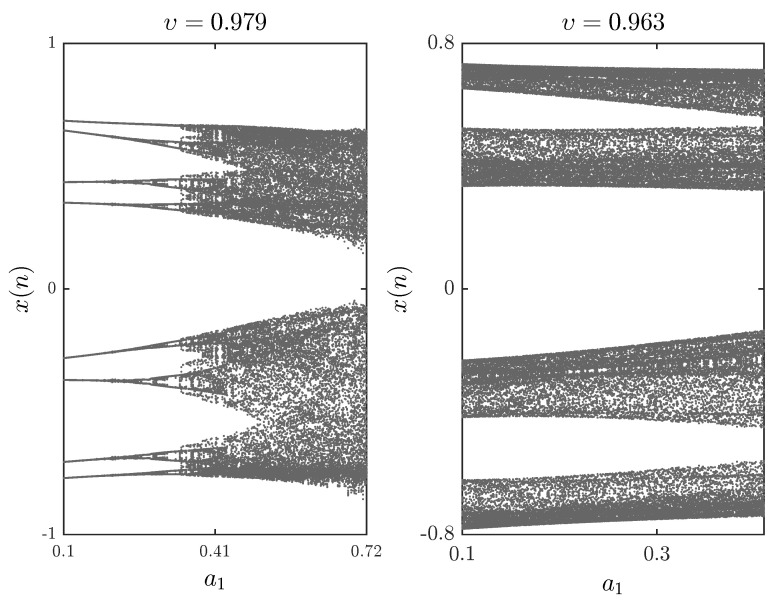
Bifurcation diagrams with $a_{1}$ as the critical parameter and $\left(a_{2},a_{3},a_{4}\right)=\left(1,1.51,0.74\right)$ and $\left(x(0),y(0)\right)=\left(-0.81,0.51\right)$ for different fractional orders υ.

**Figure 7:**
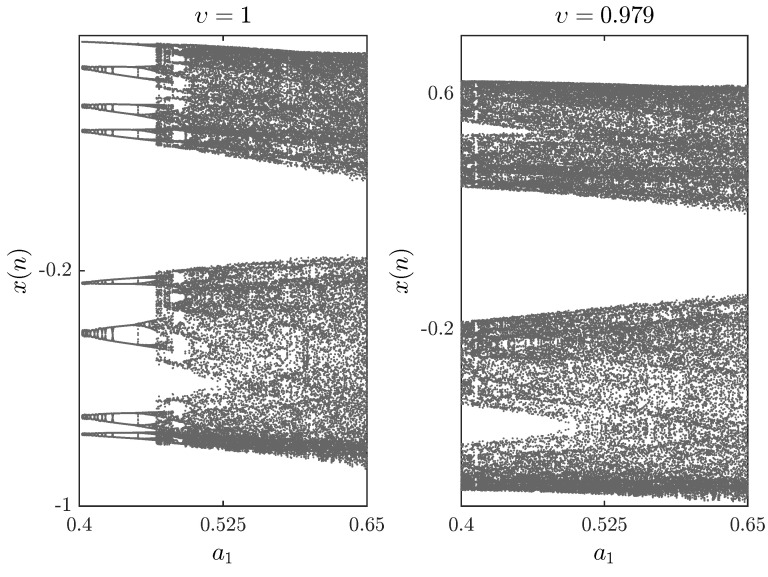
Bifurcation diagrams with $a_{1}$ as the critical parameter and $\left(a_{2},a_{3},a_{4}\right)=\left(1,1.6,0.72\right)$ and $\left(x(0),y(0)\right)=\left(-0.26,0.18\right)$ for different fractional orders υ.

**Figure 8:**
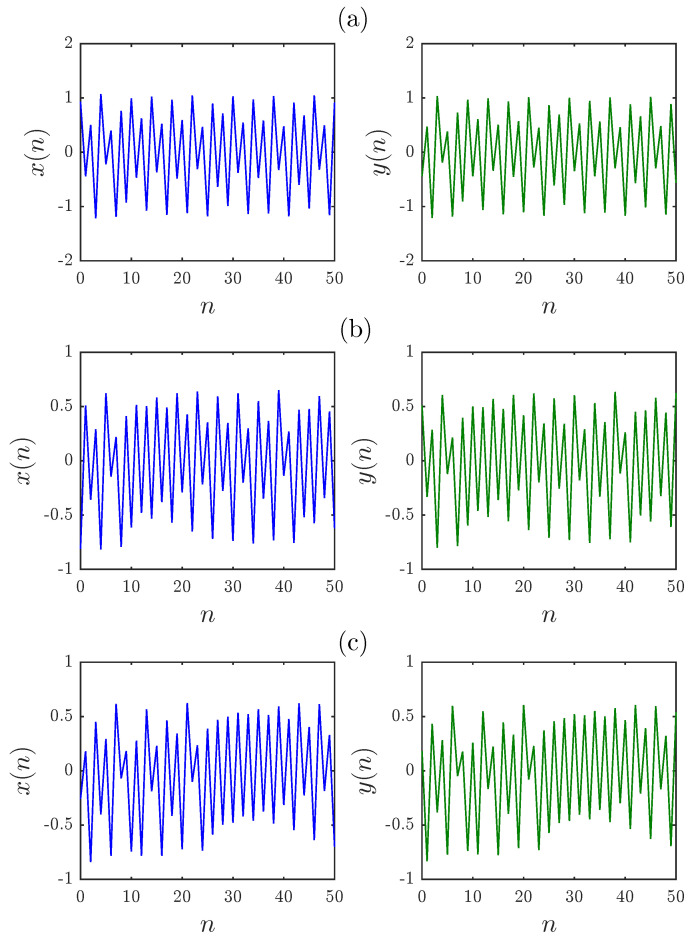
Time evolution of states for υ=0.979 and: (**a**) $\left(a_{1},a_{2},a_{3},a_{4}\right)=\left(0.2,0.71,0.91,1.14\right)$ and $\left(x(0),y(0)\right)=\left(0.93,-0.44\right)$; (**b**) $\left(a_{1},a_{2},a_{3},a_{4}\right)=\left(0.51,1,1.51,0.74\right)$ and $\left(x(0),y(0)\right)=\left(-0.81,0.51\right)$; (**c**) $\left(a_{1},a_{2},a_{3},a_{4}\right)=\left(0.6,1,1.6,0.72\right)$ and $\left(x(0),y(0)\right)=\left(-0.26,0.18\right)$.

The bifurcation diagrams for different parameters $\left(a_{1},a_{2},a_{3},a_{4}\right)$ are shown in Figure 5, Figure 6 and Figure 7, respectively. First we fix parameters $\left(a_{1},a_{3},a_{4}\right)$–$\left(0.2,0.91,1.14\right)$ and vary $a_{2}$ along the interval $\left[0.46,0.75\right]$. Clearly, decreasing the fractional order υ affects the interval over which chaos is exhibited. In Figure 6, the bifurcation diagram is obtained with $\left(a_{2},a_{3},a_{4}\right)=\left(1,1.51,0.74\right)$ and the critical parameter $a_{1}$ being varied in steps of $\Delta a_{1}=0.0006$. In this case, when we decrease the fractional order υ, the opposite is observed as the chaotic band expands and the eight-period stage disappears. Finally, Figure 7 is obtained for $\left(a_{2},a_{3},a_{4}\right)=\left(1,1.6,0.72\right)$ with $a_{1\;}$ as the critical parameter. We see that a slight change in the fractional order has a considerable effect on the dynamics of the fractional map. For completeness, the time evolution of the states belonging to the fractional map are displayed in Figure 8 for υ=0.979.

### 3.2. Entropy Analysis

In information theory, entropy is a logarithmic measure that quantifies the rate of transfer or generation of information in a particular system. For discrete-time dynamical systems in general, Kolmogorov–Sinai (KS) entropy is an interesting measure. A direct time-series approximation of the KS entropy was developed in [42], termed Eckmann–Ruelle (ER) entropy, which quickly became appealing as a way of quantifying the level of chaos present in a particular system. The idea is that instead of looking at the phase plots of bifurcation diagrams, an exact measure of the information generated in a sequence is more indicative of the level of chaos. Calculating the exact ER entropy experimentally is impossible. Rather, an approximate entropy (ApEn) measure was proposed in [43,44]. ApEn has been used extensively in the literature to investigate chaos in discrete dynamical systems [25,45]. In order to examine the level of chaotic behavior present in the fractional map (6), we have measured its approximate entropy (ApEn) and listed it in Table 1. We have calculated the approximate entropy using the known algorithm [43,44]. As can be seen from Table 1, the results match with the ones shown in Figure 2 and Figure 3. For the set of parameters $\left(a_{1},a_{2},a_{3},a_{4}\right)=\left(0.2,0.71,0.91,1.14\right)$ and $\left(x(0),y(0)\right)=\left(0.93,-0.44\right)$, the complexity of the fractional map without a fixed point is increased when reducing the value of different fractional order υ. The complexity of the fractional map without a fixed point changes when decreasing the value of υ for $\left(a_{1},a_{2},a_{3},a_{4}\right)=\left(0.51,1,1.51,0.74\right)$ and $\left(x(0),y(0)\right)=\left(-0.81,0.51\right)$.

## 4. Stabilization Control

In this section, we aim to propose a one-dimensional control law that stabilizes the states of our fractional map (6). Stabilization refers to the adaptive control of one or more system states to ensure all of the states converge asymptotically towards an equilibrium point. In our case, we assume the equilibrium to be the all zero state. Before we present our result, we recall an important theorem related to the asymptotic stability of discrete fractional systems through system linearization.

**Theorem** **2**  ([37])**.** *Given a vector-valued function $X\left(t\right)={\left(x_{1}\left(t\right),\ldots ,x_{n}\left(t\right)\right)}^{T}$, 0<υ≤1, $A\in R^{n\times n}$ and $\forall t\in N_{a+1-\upsilon }$, the zero equilibrium of system:*

(13) $${}^{C}\Delta_{a}^{\upsilon }X\left(t\right)=AX\left(t+\upsilon -1\right),$$

*is asymptotically stable if:*

(14) $$\lambda \in \left\{z\in C:\;\left|z\right|<{\left(2\cos\frac{\left|\arg z\right|-\pi }{2-\upsilon }\right)}^{\upsilon }\;and\;\left|\arg z\right|>\frac{\upsilon \pi }{2}\right\},$$

*for all the eigenvalues λ of A.*

Theorem 2 will help us establish the asymptotic convergence of our stabilized system states to zero. The following theorem summarizes our result.

**Theorem** **3.** *The 2D fractional map (6) can be controlled under the 1D control law:*

(15) $$u\left(t\right)=-x\left(t\right)-a_{1}x^{2}\left(t\right)-a_{2}y^{2}\left(t\right)+a_{3}x\left(t\right)y\left(t\right)+a_{4}.$$

**Proof.**  Adding a time-varying control term $u\left(t\right)$ to the second state of our fractional map (6) yields:

(16) $$\left\{\begin{array}{cc} {}^{C}\Delta_{a}^{\upsilon }x\left(t\right) & =y\left(t-1+\upsilon \right)-x\left(t-1+\upsilon \right),\\ {}^{C}\Delta_{a}^{\upsilon }y\left(t\right) & =x\left(t-1+\upsilon \right)+a_{1}x^{2}\left(t-1+\upsilon \right)+a_{2}y^{2}\left(t-1+\upsilon \right)\\ & \;-a_{3}x\left(t-1+\upsilon \right)y\left(t-1+\upsilon \right)-a_{4}-y\left(t-1+\upsilon \right)\\ & \;+u\left(t+\upsilon -1\right). \end{array}\right.$$

The aim is to show that with $u\left(t\right)$ defined according to (15), the states of (16) converge towards zero asymptotically. In other words, we want to show that point $\left(0,0\right)$ in phase-space is an asymptotically stable equilibrium of the system resulting from substitution of (15) into (16), which is simply:

(17) $$\left\{\begin{array}{c} {}^{C}\Delta_{a}^{\upsilon }x\left(t\right)=-x\left(t-1+\upsilon \right)+y\left(t-1+\upsilon \right),\\ {}^{C}\Delta_{a}^{\upsilon }y\left(t\right)=-y\left(t-1+\upsilon \right). \end{array}\right.$$

System (17) is linear, which makes our job easy. We write it in matrix form as:

(18) $${}^{C}\Delta_{a}^{\upsilon }\left(\begin{array}{c} x\left(t\right)\\ y\left(t\right) \end{array}\right)=A\left(\begin{array}{c} x\left(t\right)\\ y\left(t\right) \end{array}\right),$$

where:

(19) $$A=\left(\begin{array}{cc} -1 & 1\\ 0 & -1 \end{array}\right).$$

Since A is upper-triangular, its eigenvalues are simply $\lambda_{1}=\lambda_{2}=-1$, and both of them satisfy:

(20) $$\left|\lambda_{i}\right|<{\left(2\cos\frac{\left|\arg\lambda_{i}\right|-\pi }{2-\upsilon }\right)}^{\upsilon }\;\mathrm{and}\;\left|\arg\lambda_{i}\right|>\frac{\upsilon \pi }{2},\;\;i=1,2.$$

Hence, by Theorem 2, we know that the zero equilibrium of (17) is asymptotically stable, and consequently, the states of (16) are stabilized. ☐

**Figure 9:**
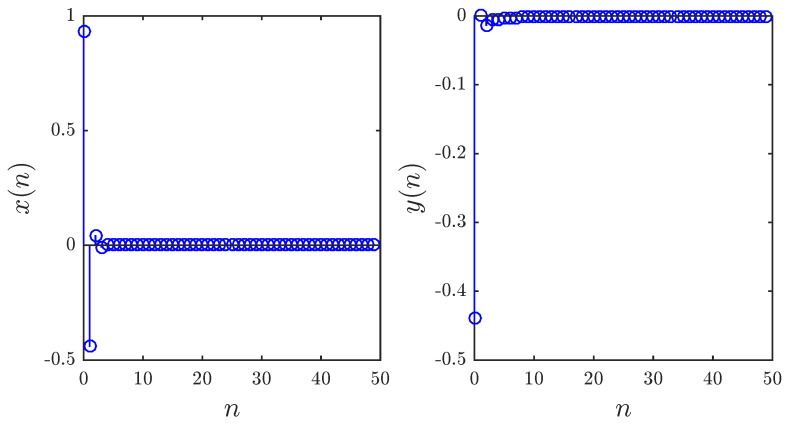
Stabilized states of the fractional map based on control law (15) with $\left(x\left(0\right),y\left(0\right)\right)=\left(0.93,-0.44\right)$, $\left(a_{1},a_{2},a_{3},a_{4}\right)=\left(0.2,0.71,0.91,1.14\right)$ and υ=0.97.

The control strategy proposed in Theorem 3 has been implemented numerically to confirm its validity. The fractional map proposed in this paper was run with initial conditions $\left(x\left(0\right),y\left(0\right)\right)=\left(0.93,-0.44\right)$, parameters $\left(a_{1},a_{2},a_{3},a_{4}\right)=\left(0.2,0.71,0.91,1.14\right)$ and fractional order υ=0.97. The term $u\left(t\right)$ defined in (15) was computed iteratively based on previous states and added to the new second state. Figure 9 depicts the time evolution of the states. Clearly, the states converge towards zero, and thus, our stabilization is successful.

**Figure 10:**
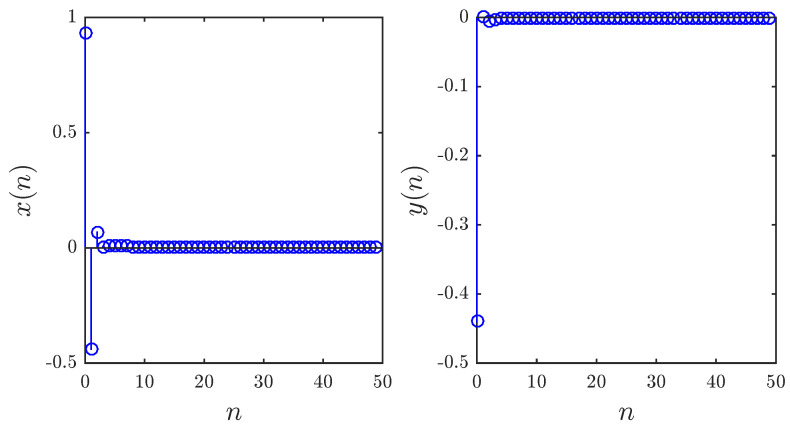
Stabilized states of the fractional map based on control law (15) with $\left(x\left(0\right),y\left(0\right)\right)=\left(0.93,-0.44\right)$, $\left(a_{1},a_{2},a_{3},a_{4}\right)=\left(0.2,0.71,0.91,1.14\right)$ and $\left(\upsilon_{x},\upsilon_{y}\right)=\left(0.95,0.99\right)$.

**Figure 11:**
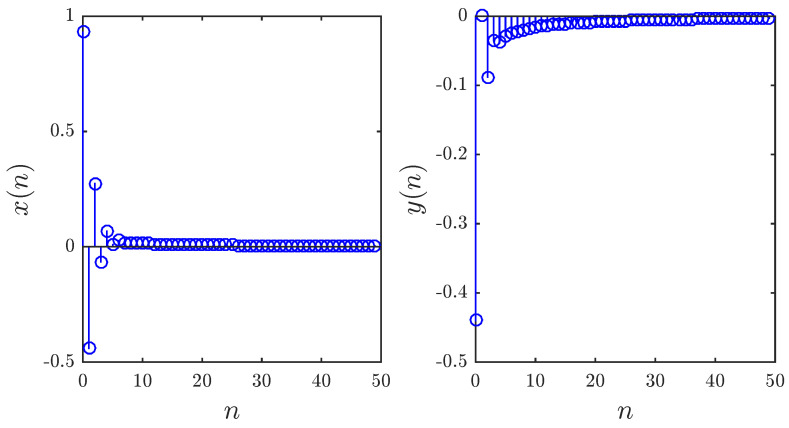
Stabilized states of the fractional map based on control law (15) with $\left(x\left(0\right),y\left(0\right)\right)=\left(0.93,-0.44\right)$, $\left(a_{1},a_{2},a_{3},a_{4}\right)=\left(0.2,0.71,0.91,1.14\right)$ and time varying fractional order (21).

Note that the asymptotic convergence of the control law proposed in Theorem 3 was only established in the commensurate case, i.e., identical fractional orders for all states. However, experimental results have in fact shown that the zero solution of the feedback controlled system is asymptotically stable over a range of circumstances including the incommensurate case and the time varying fractional order case. Figure 10 shows the stabilized states subject to the same parameters and initial setting adopted earlier, but with fractional orders $\left(\upsilon_{x},\upsilon_{y}\right)=\left(0.95,0.99\right)$. The convergence of the states towards zero is apparent. Figure 11 depicts the time evolution of the controlled states when the fractional order is a discrete function of time given by:

(21) $$\upsilon \left(t\right)=0.8+0.2\sin\left(\frac{x}{50}\right).$$

Again, the states converge in a very steady way towards zero, but as expected, take longer to converge compared to the standard case.

## 5. Discussion and Conclusions

In this paper, we have examined a fractional chaotic map based on the standard generic map proposed in [14], which exhibits rich dynamics and hidden chaotic attractors under different circumstances, i.e., with no fixed points, with a single fixed point or with two fixed points. Using phase-space portraits and bifurcation diagrams, we have shown that varying the fractional order impacts the parameter interval over which chaos is observed, as well as the shape of the resulting attractors. We have also quantified the level of chaos present in the proposed map by means of the approximate entropy measure. In addition, we have presented a one-dimensional stabilization controller that forces the system states towards zero asymptotically. Numerical methods were employed to confirm the convergence of this controller under different scenarios. The controller has been shown to be resilient to the time variation of the fractional order. As mentioned before, the standard map upon which our proposed system is based exhibits hidden attractors. This feature has not been assessed for the new fractional map. It is our intention to address this point in a future study.

In addition, it has been claimed in many studies that fractional chaotic maps are superior to their integer counterparts as they involve new degrees of freedom due to the dependence of the maps’ dynamics and trajectory on the fractional order [32]. This makes them of particular importance in fields where the seemingly random nature of the dynamical states is a desired property such as in data and image encryption [28]. For instance, we believe that by varying the fractional order by means of some deterministic time series, a new dimension can be introduced into the encryption process, making it harder to break. We have not seen any such studies in the literature. Perhaps the reason for that is the fact that it is rather difficult to establish the convergence of the synchronization errors in such a scenario. However, experiments have shown that many of the proposed controllers in the literature remain valid when the fractional order varies in the same way at the master and slave sides. This will be investigated thoroughly in a future study.

## Figures and Tables

**Table 1:** Approximate entropy calculation of the fractional map for different values of parameters and υ.

Case	υ	ApEn
Figure 2	1	0.0903
Figure 2	0.985	0.0955
Figure 2	0.979	0.1094
Figure 3	1	0.2083
Figure 3	0.979	0.1766
Figure 3	0.963	0.2184
